# Enhanced Recovery After Surgery in Patients With Hepatocellular Carcinoma Undergoing Laparoscopic Hepatectomy

**DOI:** 10.3389/fsurg.2021.764887

**Published:** 2021-11-22

**Authors:** Jiamin Zhou, Xigan He, Miao Wang, Yiming Zhao, Ning Zhang, Longrong Wang, Anrong Mao, Lu Wang

**Affiliations:** ^1^Department of Hepatic Surgery, Shanghai Cancer Center, Fudan University, Shanghai, China; ^2^Department of Oncology, Shanghai Medical College, Fudan University, Shanghai, China

**Keywords:** enhanced recovery after surgery, hepatocellular carcinoma, hepatectomy, laparoscopy, liver function

## Abstract

**Objective:** To compare the effectiveness and safety of enhanced recovery after surgery (ERAS) in patients with hepatocellular carcinoma (HCC) undergoing laparoscopic hepatectomy.

**Methods:** From September 2016 to June 2019, 282 patients were enrolled, and ERAS was implemented since March 2018. All indicators related to surgery, liver function, and postoperative outcomes were included in the analysis. Propensity score matching (PSM) identified 174 patients for further comparison.

**Results:** After PSM, the clinicopathological baselines were well-matched. The group showed significantly less intraoperative blood loss (100.00 [100.00–200.00] vs. 200.00 [100.00–300.00] ml, *P* = 0.001), fewer days before abdominal drainage tube removal (4.00 [3.00–4.00] days vs. 4.00 [3.00–5.00] days, *P* = 0.023), shorter hospital stay after surgery (6.00 [5.00–6.00] days vs. 6.00 [6.00–7.00] days, *P* < 0.001), and reduced postoperative morbidity (18.39 vs. 34.48%, *P* = 0.026). The proportion of patients with a pain score ≥ 4 was significantly lower in the ERAS group within the first 2 days after surgery (1.15 vs. 13.79% and 8.05 vs. 26.44%, *P* = 0.002 and *P* = 0.001, respectively). Pringle maneuver was performed more frequently in the ERAS group (70.11 vs. 18.39%, *P* < 0.001), and a significantly higher postoperative alanine aminotransferase level was also observed (183.40 [122.85–253.70] vs. 136.20 [82.93–263.40] U/l, *P* = 0.026). The 2-year recurrence-free survival was similar between the two groups (72 vs. 71%, *P* = 0.946).

**Conclusions:** ERAS programs are feasible and safe and do not influence mid-term recurrence in HCC patients undergoing laparoscopic hepatectomy.

## Introduction

Since enhanced recovery after surgery (ERAS) programs were introduced by Kehlet in the 1990's, they have been widely applied in gastrointestinal, urologic, gynecological, orthopedic surgery, and many other surgical fields to minimize perioperative pain and stress, reduce morbidity, and accelerate postoperative recovery worldwide (1–11). Furthermore, the concept of ERAS is constantly being updated with continuous clinical practice (4).

Although the implementation of ERAS programs in hepatic surgery was slightly later than in other surgical fields (12), many randomized controlled trials (RCTs), meta-analyses, and guidelines and consensus have been performed or established specifically focusing on hepatectomy (13–20). However, most of the studies have only focused on the hepatectomy itself, while the type of liver tumors and the type of surgical approach used in these studies were always mixed.

Hepatocellular carcinoma (HCC) is still one of the leading causes of cancer-related mortality, especially in China. Most cases of hepatitis B virus (HBV)-related HCC occur in patients with cirrhosis (21, 22). In several previous studies, ERAS programs were considered to be beneficial to HCC patients, especially in patients with cirrhosis that may partly be attributed to the omission of overnight fasting and carbohydrate loading, which may lessen the nutritional stress (23, 24). However, only a few studies have focused on this field.

Since the second international consensus conference for laparoscopic liver resection in 2014, laparoscopic minor hepatectomy was the standard surgical practice (25). In addition, laparoscopic major hepatectomy was gradually accepted for its safety, feasibility, and good short- and long-term outcomes, including in HCC patients with cirrhosis in recent years (26, 27). Although many previous studies have explored the recovery of patients undergoing both ERAS programs and laparoscopic hepatectomy (LH), their results did not seem to be consistent (16, 23, 28, 29). The results might also be due to the mismatch of the type of liver tumors and the ratio of LH among these studies. Thus, it is meaningful to focus on the role of ERAS programs in patients with HCC undergoing LH.

Therefore, this study aimed to compare the effectiveness and safety of ERAS in patients with HCC undergoing LH.

## Materials and Methods

The inclusion criteria were as follows: (1) patients were pathologically confirmed to have HCC after surgery; (2) all surgical procedures were successfully performed by laparoscopy; (3) radical resection was achieved; and (4) preoperative liver function was Child-Pugh A or B. The exclusion criteria were as follows: (1) patients underwent laparoscopic radiofrequency ablation alone, and (2) laparoscopic surgery was converted to open surgery for any reason. From September 2016 to June 2019, 282 eligible patients in the Department of Hepatic Surgery, Shanghai Cancer Center, Fudan University, were enrolled in the study retrospectively. ERAS programs were implemented in our center on March 1, 2018. All the patients who were suitable for undergoing LH routinely followed the ERAS protocol. Therefore, 108 patients from September 2016 to February 2018 were enrolled in the control group, and the subsequent 174 patients were enrolled in the ERAS group. All surgical operations were performed by the team of Prof. Lu Wang. This study was approved by the Research Ethics Committee of Shanghai Cancer Center, Fudan University.

Enhanced recovery after surgery (ERAS) management measures at our center were introduced in our previous study (30) and are briefly described in Table 1. The underlying diseases of the patients were defined as cardiovascular disease, cerebrovascular disease, hypertension, diabetes, and other chronic diseases, namely, chronic bronchitis, chronic kidney disease, and rheumatoid arthritis. HBV infection referred to patients with HBsAg positivity, regardless of whether the HBV DNA was replicating or not. The tumor stage was defined according to the Barcelona Clinic Liver Cancer (BCLC) staging. Postoperative complications were defined and classified according to the Clavien–Dindo classification criteria. The pain score was classified according to the visual analog scale, and a score ≥ 4 was defined as severe pain requiring analgesic treatment. Four liver function-related indicators, namely, alanine aminotransferase (ALT), total bilirubin (TB), prothrombin time (PT), and prealbumin (PAB), were used to express the postoperative recovery of liver function, all of which were recorded before the surgery and on the 3rd day after surgery. Abdominal contrast-enhanced computed tomography scanning or magnetic resonance imaging, serum alpha-fetoprotein levels, and chest radiographs were monitored at an interval of 3 months after liver resection within the first 2 years. Recurrence-free survival (RFS) was defined as the interval between surgery and recurrence. If recurrence was not diagnosed, patients were censored on the date of death or the last follow-up. Two years was generally set as the cutoff value to define early recurrence (31).

**Table 1:** Enhanced recovery after surgery (ERAS) programs in our center.

	**ERAS programs**
Preoperative management	ERAS programs are introduced during preoperative education
	NRS-2002 evaluation scale is used to determine preoperative nutritional assessment and support
	No preoperative bowel preparation
	Patients were fasted for 6 h and drink was forbidden for 2 h before surgery
	Child-Pugh liver function grading evaluation
	Accurate liver resection planning under three-dimensional reconstruction and ERAS management risk evaluation and control
	Routine evaluation and prevention training, focusing on the risk evaluation of deep venous thrombosis and respiratory function exercise
Intraoperative management	Routine usage of prophylactic antibiotics
	Multi-mode individualized anesthesia program
	Low central venous pressure (CVP) technique [CVP <5 mmHg, systolic blood pressure (SBP) > 90 mmHg] + perioperative goal directed fluid therapy
	Individualized liver blood flow control technique
	Perioperative body temperature higher than 36.0°C (insulation blanket + warm distilled water rinse)
	Open/laparoscopy + delicate liver parenchyma dissection technique
Postoperative management	Selective indwelling drainage tube, no routine nasogastric tube, early removal of catheters
	Comprehensive, quantitative and dynamic evaluation + preventive multi-mode analgesic management (routine analgesic pump 1d, supplemented by opioids, non-steroidal anti-inflammatory drugs and epidural anesthesia)
	PONV risk evaluation and multi-mode PONV prevention (such as 5-HT3 receptor antagonist and glucocorticoid)
	Patients were encouraged to drink water 4–6 h after surgery and to take a liquid or semi-liquid diet 1 d after surgery, gradually to a normal diet
	Mobilization was started at 1 d after surgery. Establish daily activity goals and increase activity levels gradually.
	In addition to routine care and symptomatic treatment, focusing on coagulation dysfunction (routine low-molecular heparin)/liver failure/bile leakage/ascites/hydrothorax and other complications
	Discharge as soon as possible in accordance with the criteria: basic self-care; pain relief or oral pain relievers can control pain well; normally diet without intravenous fluids support; normal flatus and defecation; the Child-Pugh liver function grade A or bilirubin returned to normal or nearly normal; good wound healing and no infection; no need to wait for removing stitches; the patient agreed and wished to be discharged.

*PONV, Postoperative nausea and vomiting*.

Propensity score matching (PSM) analysis was performed in this study to reduce bias in patient selection using SPSS 22.0 for Windows (SPSS Inc., Chicago, IL, USA). The variables in the clinicopathological baseline that were not balanced and might affect the results, namely, age, sex, underlying diseases, HBV infection, American Society of Anesthesiologists (ASA) score, body mass index (BMI), preoperative level of ALT, TB, PT, PAB, and type of hepatectomy were included in the calculation. The propensity score was generated using logistic regression with these variables, and the caliper value was set to 0.02. The patients were selected using nearest-neighbor matching without replacement at a ratio of 1:1. A two-sample Student's *t*-test or Mann–Whitney *U*-test was performed to compare quantitative variables. For data analyzed with a two-sample Student's *t*-test, the data were presented as mean ± standard error, and for data analyzed with Mann–Whitney *U* test, the data were presented as median (interquartile range). Pearson's χ^2^ test or Fisher's exact test was used to comparing qualitative variables. Statistical analyses were performed using the SPSS 22.0. Plot analysis was performed using GraphPad Prism 5 for Windows (GraphPad Software, Inc. San Diego, CA, USA). Statistical significance was set at *P* < 0.05.

## Results

A total of 282 patients were recruited for this study. Among these, 108 patients (38.30%) received traditional perioperative care in the control group, and 174 patients (61.70%) received ERAS programs in the ERAS group. The clinicopathological characteristics of these cohorts are summarized in Table 2. The sex, HBV infection, BMI, preoperative TB, PT, PAB, type of hepatectomy, and BCLC stage of patients in these two groups were balanced. However, a significantly higher proportion of elderly patients (*P* = 0.025), patients with underlying diseases (*P* = 0.010), higher ASA scores (*P* = 0.017), and lower preoperative ALT levels (*P* = 0.032) were observed in the ERAS group.

**Table 2:** The clinicopathological characteristics of patients.

	**Before propensity score matching**			**After propensity score matching**		
**Variables**	**Control group (*n* = 108)**	**ERAS group (*n* = 174)**	***P***	**Control group (*n* = 87)**	**ERAS group (*n* = 87)**	***P***
Age, years	54.00 (47.00–62.50)	59.00 (49.00–65.00)	**0.025**	55.22 ± 1.09	54.89 ± 1.28	0.843
**Sex, %**			0.732			0.463
Male	87 (80.56%)	143 (82.18%)		70 (80.46%)	66 (75.86%)	
Female	21 (19.44%)	31 (17.82%)		17 (19.54%)	21 (24.14%)	
**Underlying diseases, %**			**0.010**			0.339
Yes	29 (26.85%)	73 (41.95%)		27 (31.03%)	33 (37.93%)	
No	79 (73.15%)	101 (58.05%)		60 (68.97%)	54 (62.07%)	
**HBV infection, %**			0.521			0.517
Yes	90 (83.33%)	146 (83.91%)		76 (87.36%)	73 (83.91%)	
No	18 (16.67%)	28 (16.09%)		11 (12.64%)	14 (16.09%)	
ASA score^*^, %			**0.017**			0.091
I	31 (29.81%)	33 (19.76%)		24 (27.59%)	20 (22.99%)	
II	65 (62.50%)	129 (77.25%)		55 (63.22%)	65 (74.71%)	
III	8 (7.69%)	4 (2.40%)		8 (9.20%)	2 (2.30%)	
IV	0 (0.00%)	1 (0.60%)		-	-	
BMI	23.78 ± 0.32	24.03 ± 0.25	0.553	23.74 ± 0.34	23.93 ± 0.35	0.703
**Preoperative indicators**						
ALT, U/l	29.60 (19.90–43.45)	25.10 (17.55–34.95)	**0.032**	27.90 (19.30–39.80)	26.70 (17.60–35.40)	0.519
TB, μmol/l	13.00 (9.90–16.45)	11.60 (8.90–15.15)	0.050	12.90 (9.20–16.20)	12.20 (9.60–16.60)	0.892
PT, s	13.40 (13.05–14.15)	13.30 (12.85–14.00)	0.059	13.40 (13.10–14.00)	13.30 (12.80–14.10)	0.442
PAB, mg/l	238.00 (206.00–277.00)	241.00 (206.50–287.50)	0.240	241.00 (206.00–277.00)	238.00 (197.00–277.00)	0.813
**Type of hepatectomy, %**			0.599			0.341
Extensive hemihepatectomy	0 (0.00%)	2 (1.15%)		-	-	
Hemihepatectomy	6 (5.56%) left 5, right 1	9 (5.17%)left 5, right 4		5 (5.75%)left 5	1 (1.15%) left 1	
Segmentectomy	46 (42.59%) VII 4, right posterior 6	84 (48.28%)I 1, VII 4, right posterior 7		41 (47.13%)VII 3, right posterior 6	43 (49.43%) I 1, VII 2, right posterior 2	
Local recection	56 (51.85%) VII 7	79 (45.40%)VII 21		41 (47.13%)VII 6	43 (49.43%) VII 12	
**BCLC stage, %**						
0	12 (11.11%)	17 (9.77%)		11 (12.64%)	9 (10.34%)	
A	84 (77.78%)	135 (77.59%)		65 (74.71%)	71 (81.61%)	
B	9 (8.33%)	18 (10.34%)		8 (9.20%)	7 (8.05%)	
C	3 (2.78%)	4 (2.30%)		3 (3.45%)	0 (0.00%)	

*HBV, Hepatitis B virus; ASA, American society of anestheiologists; BMI, Body mass index; ALT, Alanine aminotransferase; TB, Total bilirubin; PT, Prothrombin time; PAB, Prealbumin; BCLC, Barcelona Clinic Liver Cancer*.

* *partial value of ASA score was missing. The bold P values were used to highlight the variables with P <0.05*.

The operative results and postoperative outcomes in the entire patient population are shown in Table 3. As for the operation-related indicators, significantly less intraoperative blood loss was observed in the ERAS group (200.00 [100.00–400.00] vs. 175.00 [100.00–275.00] ml, *P* = 0.009). Although the proportion of intraoperative blood transfusion was similar (5.56 vs. 3.45%, *P* = 0.545) in the two groups, the type of hepatectomy was different (control group: segmentectomy 3 and local resection 3 vs. ERAS group: extensive hemihepatectomy 1, segmentectomy 4, and local resection 1). The use of the Pringle maneuver was also significantly more frequent in the ERAS group (66.10 vs. 17.59%, *P* < 0.001). As an indicator of postoperative liver function recovery, the TB level was significantly lower in the ERAS group (24.00 [18.00–35.00] vs. 27.25 [22.68–41.63] μmol/l, *P* = 0.002), while the ALT, PT, and PAB levels showed no significant difference between these two groups. The postoperative outcomes, namely, the days that semiliquid diet was allowed after surgery and hospital stay after surgery, were significantly less in the ERAS group (2.00 [2.00–2.00] days vs. 3.00 [3.00–4.00] days and 6.00 [5.00–6.00] days vs. 6.00 [6.00–7.00] days, both *P* < 0.001). In terms of pain score, the proportion of patients with a score ≥ 4 was significantly lower in the ERAS group within the first 2 days after surgery (2.87 vs. 12.96% and 9.77 vs. 24.07%, both *P* < 0.001). There was no significant difference in the abdominal drainage tube indwelling duration and the hospital costs between these two groups were also similar (47,069.39 [40,980.86–54,488.74] CNY vs. 49,498.55 [42,812.30–57,936.92] CNY, *P* = 0.158). The incidence of complications was not significantly different between the two groups (25.29 vs. 36.11%, *P* = 0.182). Furthermore, the 2-year RFS was similar between the two groups (71 vs. 72%, *P* = 0.887).

**Table 3:** The operative results and postoperative outcomes.

	**Before propensity score matching**			**After propensity score matching**		
**Variables**	**Control group (*n* = 108)**	**ERAS group (*n* = 174)**	***P***	**Control group (*n* = 87)**	**ERAS group (*n* = 87)**	***P***
Operative duration, mins	128.00 (100.00–174.25)	126.50 (101.25–170.00)	0.259	125.50 (96.25–176.50)	121.50 (98.50–163.00)	0.127
Blood loss, ml	200.00 (100.00–400.00)	175.00 (100.00–275.00)	**0.009**	200.00 (100.00–300.00)	100.00 (100.00–200.00)	**0.001**
**Intraoperative blood transfusion, %**			0.545			0.682
Yes	6 (5.56%) Segmentectomy 3, local resection 3	6 (3.45%)Extensive hemihepatectomy 1, Segmentectomy 4, local resection 1		4 (4.60%)Segmentectomy 2, local resection 2	2 (2.30%) Segmentectomy 1, local resection 1	
No	102 (94.44%)	168 (96.55%)		83 (95.40%)	85 (97.70%)	
**Pringle maneuver**			** <0.001**			** <0.001**
Yes	19 (17.59%)	115 (66.10%)		16 (18.39%)	61 (70.11%)	
No	89 (82.41%)	59 (33.91%)		71 (81.61%)	26 (29.89%)	
**Postoperative indicators**						
ALT, U/l	146.15 (85.18–254.48)	174.10 (109.80–261.53)	0.195	136.20 (82.93–263.40)	183.40 (122.85–253.70)	**0.026**
TB, μmol/l	27.25 (22.68–41.63)	24.00 (18.00–35.00)	**0.002**	27.25 (22.85–38.78)	24.90 (18.10–35.38)	0.073
PT, s	14.90 (14.40–15.70)	14.75 (14.00–15.68)	0.935	14.90 (14.40–15.68)	14.90 (14.20–15.68)	0.649
PAB, mg/l	126.57 ± 3.15	128.41 ± 2.88	0.677	125.74 ± 3.38	126.41 ± 3.79	0.896
**Pain score** **≥** **4, yes/no, %**						
POD 1	14/94 (12.96%)	5/169 (2.87%)	**0.001**	12/75 (13.79%)	1/86 (1.15%)	**0.002**
POD 2	26/82 (24.07%)	17/157 (9.77%)	**0.001**	23/64 (26.44%)	7/80 (8.05%)	**0.001**
POD 3	12/96 (11.11%)	9/165 (5.17%)	0.065	10/77 (11.49%)	4/83 (4.60%)	0.094
POD 4	7/101 (6.48%)	6/168 (3.45%)	0.255	5/82 (5.75%)	4/83 (4.60%)	1.000
POD 5	2/106 (1.85%)	0/174 (0.00%)	0.146	2/85 (2.30%)	0/87 (0.00%)	0.497
Semiliquid diet after surgery, days	3.00 (3.00–4.00)	2.00 (2.00–2.00)	**<** **0.001**	3.00 (3.00–4.00)	2.00 (2.00–2.00)	**<** **0.001**
Abdominal drainage tube removal, days	4.00 (3.00–5.00)	4.00 (3.00–5.00)	0.053	4.00 (3.00–5.00)	4.00 (3.00–4.00)	**0.023**
Hospital stay after surgery, days	6.00 (6.00–7.00)	6.00 (5.00–6.00)	**<** **0.001**	6.00 (6.00–7.00)	6.00 (5.00–6.00)	**<** **0.001**
Hospital costs, CNY	49498.55 (42812.30–57936.92)	47069.39 (40980.86–4488.74)	0.158	49397.18 (42749.59–56975.35)	46219.98 (41353.38–51841.06)	0.123
**Complications, %**			0.182			**0.026**
No	69 (63.89%)	130 (74.71%)		57 (65.52%)	71 (81.61%)	
Grade I	18 (16.67%)	24 (13.79%)		13 (14.94%)	11 (12.64%)	
Grade II	18 (16.67%)	15 (8.62%)		15 (17.24%)	5 (5.75%)	
Grade III	3 (2.78%)	4 (2.30%)		2 (2.30%)	0 (0.00%)	
Grade IV	0 (0.00%)	1 (0.57%)		-	-	

*POD, Postoperative day. The bold P values were used to highlight the variables with P <0.05*.

After PSM, the clinicopathological baselines of the two groups were well-matched (Table 2). In the operation-related indicators, the ERAS group showed significantly less intraoperative blood loss than the control group (100.00 [100.00–200.00] vs. 200.00 [100.00–300.00] ml, *P* = 0.001). The proportion of patients with intraoperative blood transfusion was slightly more in the control group (4.60 vs. 2.30%, *P* = 0.682) and the type of hepatectomy between the two groups was similar (control group: segmentectomy 2 and local resection 2 vs. ERAS group: segmentectomy 1 and local resection 1). The operative duration and intraoperative blood transfusion did not demonstrate any obvious differences. The Pringle maneuver was performed more frequently in the ERAS group (70.11 vs. 18.39%, *P* < 0.001). Impressively, in the liver function recovery indicators, the ALT level in the ERAS group was significantly higher than that in the control group (183.40 [122.85–253.70] vs. 136.20 [82.93–263.40] U/l, *P* = 0.026), which was completely consistent with the results of our previous study (30). On the contrary, the TB level in the ERAS group was lower than that in the control group, although the difference was not significant (24.90 [18.10–35.38] vs. 27.25 [22.85–38.78] U/l, *P* = 0.073). PT and PAB levels were also similar between the two groups.

In the postoperative outcomes, the ERAS group showed significantly fewer days that a semiliquid diet was allowed (2.00 [2.00–2.00] days vs. 3.00 [3.00–4.00] days, *P* < 0.001), abdominal drainage tube removal (4.00 [3.00–4.00] days vs. 4.00 [3.00–5.00] days, *P* = 0.023), and hospital stay after surgery (6.00 [5.00–6.00] days vs. 6.00 [6.00–7.00] days, *P* < 0.001) than in the control group. Similar to that before PSM, the proportion of patients with a pain score ≥ 4 was significantly lower in the ERAS group within the first 2 days after surgery (1.15 vs. 13.79% and 8.05 vs. 26.44%, *P* = 0.002 and *P* = 0.001, respectively). In the patient population after PSM, there was also no significant difference in the hospital costs between these two groups (46,219.98 [41,353.38–51,841.06] CNY vs. 49,397.18 [42,749.59–56,975.35] CNY, *P* = 0.123). Interestingly, the ERAS group demonstrated significantly less postoperative morbidity (18.39 vs. 34.48%, *P* = 0.026) after PSM. Furthermore, after PSM, 2-year RFS was similar in these two groups (72 vs. 71%, *P* = 0.946).

## Discussion

Laparoscopic hepatectomy (LH) has been considered as a landmark development in the progression of a surgical treatment since it was gradually introduced to cure liver lesions in the 1990s (32, 33). LH was first applied to a patient with HCC in 1998 (34). The majority of HCC patients were infected with HBV (appropriate 85% of patients in this study), which caused cirrhosis or at least an inflammatory background in the liver. Thus, the surgical risk of LH correspondingly increased. Compared with open hepatectomy, LH showed better surgical safety, faster postoperative recovery, and comparable long-term survival (35). LH itself could be regarded as an ERAS approach to reduce the impact of surgery on HCC patients (20, 36, 37). The combination of LH and ERAS programs seemed to demonstrate lower postoperative morbidity and more satisfactory functional recovery than open surgery in both minor and major liver resections (37, 38), although several meta-analyses have yielded inconsistent conclusions (15, 16, 28, 29, 39).

In this study, PSM was performed to minimize the confounding bias of the baselines due to the retrospective design. Early abdominal drainage tube removal, better pain control, shorter hospital stay, and lower postoperative morbidity after ERAS were confirmed. These results proved the effectiveness of ERAS programs in patients with HCC who had undergone LH. Furthermore, alterations in postoperative liver function and mid-term recurrence were also investigated in these patients. Several representative indicators, namely, ALT, TB, and PT were selected, and these indicators generally peaked on the 3rd day after surgery. The postoperative ALT level was significantly higher in the ERAS group. Conversely, the TB level was lower in the ERAS group, although the difference was not statistically significant. The PT levels were also not affected. Similar results were also observed in LH, but not open surgery, in our previous study (30). These indicators reflected that liver function was stable and trended to recover faster. The increasing level of ALT revealed that laparoscopic surgery combined with controlled low central venous pressure (CVP) according to the ERAS programs might enhance the ischemia-reperfusion injury (IRI) of the liver. Laparoscopic pneumoperitoneum can also cause hepatic IRI as a result of the temporary decrease in blood inflow into the portal vein (40, 41). Above all, the Pringle maneuver was performed more frequently in the ERAS group. Laparoscopic Pringle maneuver combined with low CVP obviously decreased intraoperative blood loss and tended to reduce the proportion of blood transfusion, which made LH safer. Meanwhile, low inflow and easy outflow reduce the amount of residual blood in the liver, which inevitably increases the severity of IRI (42, 43). The enrollment design of this study had a chronological sequence and the appropriate laparoscopic Pringle maneuver was gradually determined and more frequently used in the development of LH in our center, which made the Pringle maneuver used more in the ERAS group. Regardless of enhanced IRI, the laparoscopic Pringle maneuver might be considered as a step in ERAS programs.

Few studies have explored the role of ERAS programs in long-term or mid-term survival. Recently, stage III gastric cancer patients undergoing laparoscopic radical gastrectomy were verified to have a survival benefit from ERAS implementation (44). In colon cancer, laparoscopic surgery combined with ERAS has a longer overall survival than open surgery combined with ERAS (45). The potential explanations are as follows: (1) reducing stress might improve antitumor immunity and (2) quick recovery reduces delayed adjuvant therapy. Two years after surgery is a significant recurrence timing of HCC, and 2-year recurrence has an obvious influence on long-term prognosis (31). Our results showed no difference in 2-year RFS between the two groups. The potential explanation is that there is no standard adjuvant therapy for HCC, and thus, the implementation of adjuvant therapy would not be affected by ERAS. At the same time, both groups in this study were laparoscopic surgery groups, in which the role of the ERAS program alone on prognosis might be limited. In fact, ERAS implementation did not improve patient survival in all tumors (46).

Generally, in HCC patients undergoing LH, ERAS programs were verified to improve postoperative recovery significantly but did not show their role in 2-year recurrence. Although hepatic IRI might be enhanced, laparoscopic Pringle maneuver combined with low CVP might make LH safer by improving intraoperative blood loss, which should be considered as a step of ERAS programs. Although PSM was used to reduce confounding bias and make the conclusion more convincing, a following prospective RCT is still necessary to further confirm the conclusion.

## Data Availability Statement

The original contributions presented in the study are included in the article/supplementary material, further inquiries can be directed to the corresponding author/s.

## Ethics Statement

The studies involving human participants were reviewed and approved by Shanghai Cancer Center, Fudan University. The patients/participants provided their written informed consent to participate in this study.

## Author Contributions

Study concept: AM and LW. Data collection: JZ and XH. First draft: JZ. Data analysis and review and editing: All authors.

## Conflict of Interest

The authors declare that the research was conducted in the absence of any commercial or financial relationships that could be construed as a potential conflict of interest.

## Publisher's Note

All claims expressed in this article are solely those of the authors and do not necessarily represent those of their affiliated organizations, or those of the publisher, the editors and the reviewers. Any product that may be evaluated in this article, or claim that may be made by its manufacturer, is not guaranteed or endorsed by the publisher.
